# Artificial intelligence - driven toxicogenomics for predictive environmental and pharmaceutical safety

**DOI:** 10.3389/fgene.2026.1858338

**Published:** 2026-10-08

**Authors:** Mohammed Monirul Islam, Sibghatullah Muhammad Ali Sangi, Girish Meravanige, Sreeharsha Nagaraja, Muhammad Shahzad Chohan, Md Khalid Anwer, Ashesh Gurung

**Affiliations:** 1 Department of Biomedical Sciences, College of Clinical Pharmacy, King Faisal University, Al-Ahsa, Saudi Arabia; 2 Basic Medical Sciences Department, College of Medicine, Dar Al Uloom University, Riyadh, Saudi Arabia; 3 Department of Pharmaceutics, College of Pharmacy, Prince Sattam Bin Abdulaziz University, Al-Kharj, Saudi Arabia; 4 Department of Pharmacology, Kathmandu Medical College, Kathmandu, Nepal

**Keywords:** adverse drug reactions, biomarker prediction, deep learning, environmental toxicology, machine learning, toxicogenomics

## Abstract

Toxicogenomics integrates high-throughput genomic technologies with toxicology to evaluate molecular responses to chemical and pharmaceutical exposures. The increasing volume and complexity of multi-omics data necessitate advanced computational approaches capable of extracting meaningful and predictive insights. In this context, artificial intelligence (AI) and machine learning (ML) techniques—including support vector machines, random forests, and deep learning architectures—are increasingly employed for early toxicity prediction and risk assessment, with reported improvements in predictive accuracy and sensitivity relative to conventional toxicological approaches. This paper explores the role of AI-driven toxicogenomics in enhancing predictive environmental and pharmaceutical safety. It highlights the application of ML-based and data-driven models for the integrative analysis of transcriptomic, proteomic, and metabolomic datasets to identify toxicity-associated biomarkers, elucidate mechanisms of action, and predict adverse drug and environmental effects prior to clinical manifestation. Notably, AI-driven models have demonstrated significant improvements in prediction accuracy, sensitivity, and scalability compared to traditional toxicological approaches, reducing reliance on animal testing and accelerating decision-making processes. Furthermore, the paper discusses the significance of AI-based toxicogenomics in drug development, environmental contaminant screening, and regulatory toxicology, while addressing key challenges such as data heterogeneity, model interpretability, and regulatory acceptance. The convergence of computer science and toxicogenomics represents a robust framework for next-generation predictive toxicology, facilitating safer pharmaceutical development, more precise environmental risk assessment, and evidence-based public health and regulatory decision-making.

## 1 Introduction

The application of toxicogenomics, which integrates toxicology and genomics, is essential to understanding the harmful effect of chemical exposure on biological systems (National Research Council (US), 2007). Scientists can determine the health and environmental impact of various chemicals by analyzing the interactions of these chemicals at a molecular level with the human genome. Toxicity testing has predominantly relied on animal experiments and chemical assays, however, this has proven to take a long time, be costly, and is ethically problematic (Virolainen et al., 2023). Consequently, alternatives that are more efficient, affordable, and ethical are increasingly being sought after. This is where artificial intelligence or AI holds transformative potential to revolutionise toxicogenomics for predictive environmental and pharmaceutical safety (Luechtefeld and Hartung, 2025).

Artificial intelligence (AI) refers to the development of computer systems capable of performing tasks that typically require human intelligence, such as pattern recognition, learning, and decision-making. AI technologies, ranging from machine learning (ML) to deep learning (DL), natural language processing (NLP), and many others have been applied widely across disciplines, such as healthcare, finance and environment to pharmaceuticals (Tonoyan and Siraki, 2024). AI is steadily integrated into data analysis pipelines in toxicogenomics for the identification of potential toxicants and predicting their impact on humans and the environment (Zhang et al., 2025a).

Using toxicogenomics, scientists learn about the molecular processes behind toxicity. This science helps identify biomarkers of toxicity exposure, understand relationships between dose and response, and identify early signs of toxicity. The genomics’ application in toxicology can help in the understanding of individual responses to a chemical by elucidating the difference in individuals (e.g., genetic variations). The method is important for enhancing the safety and efficacy of environmental and pharmaceutical products (Alexander-Dann et al., 2018).

Toxicogenomics is used in environmental safety to evaluate the effects of pollutants on ecosystems and human beings. Environmental contaminants, such as pesticides, heavy metals and other chemicals, can harm public health. Through the use of toxicogenomics, it is possible to identify populations which may be especially sensitive to environmental toxicity. By understanding the genetic basis of toxicity, it is possible to create better regulatory policy. In addition, it helps to determine safe levels of exposure and thus minimize the risk of harm (Martins et al., 2019).

Toxicogenomics in a pharmaceutical sector drug development and safety evaluation are equally important. Drug abuse toxicity is a major reason behind the removal of drugs from the market as well as a major problem of drug research. This toxicogenomic technology can identify genes and pathways involved in adverse drug reactions (ADRs). This will predict ADRs before clinical trials. Detecting possible drug-related harm early will prevent costly failures in drug development and benefit patients (Pandiri et al., 2023).

The use of AI in toxicogenomics will help us utilize toxicity tests and safety assessments more efficiently. With the help of AI, it is possible to analyze large amounts of genomic, transcriptomic, proteomic, and metabolomic data. It identifies patterns and relations. They would be hard or impossible to identify by humans. Using these technologies can enable quick processing of bulky datasets and faster and accurate toxicity predictions (Recio-Vega et al., 2023).

The development of predictive models is among the key ways in which AI aids toxicogenomics. Toxicity prediction using machine learning refers to the use of algorithms and computer models to predict whether a substance is toxic or safe using machine learning. For example, support vector machines (SVMs), random forests, and neural networks trained on large datasets of genomic and chemical exposure data to learn the patterns of toxicity (Seal et al., 2025). When using these models, you can learn how a chemical interacts with biological systems, what effects it might have on humans and animals, and how likely it is to toxicants inducing negative health effects. AI-backed predictive models improve the efficiency of safety assessments and lessen the demand for animal testing in ways that reflect ethical and regulatory expectations (Guo et al., 2023).

Deep learning is a sub-discipline of AI that facilitates the building of more complex models that can analyze large-scale genomic data and detect patterns. Deep neural networks can handle multiple layers of data and make toxicity predictions more accurate. AI-driven toxicogenomics could develop robust models that predict a chemical’s potential for toxicity using its chemical properties, genomic sequences, and previous toxicology study data. This is possible with diverse dataset training (Mathema et al., 2023).

As another branch of AI, Natural language processing (NLP), can aid in extracting useful information from the ever-growing literature. Variety of formats are used to publish toxicological studies, clinical trials and environmental reported full of at times complex unstructured data NLP algorithms can assess this text data to extract relevant findings so that researchers can keep up with what is currently happening in toxicogenomics. The algorithms can also be used to discover new toxicity biomarkers, identify novel pathways and expedite predictive model development (Juhn and Liu, 2020).

The use of AI in toxicogenomics is valuable in environmental safety for predicting the effects of an extensive range of chemicals and contaminants on human and environmental health. The chemical composition, exposure levels, environmental data and hundreds of victim genomes inform environmental safety assessments. However, the quantity and complexity of available information is often substantial. AI algorithms can handle these multidimensional datasets appropriately, giving us insight into the potential ecological and human health risks from exposure to various pollutants (Lin and Chou, 2022).

Scientists make use of machine learning models to understand the impact of long exposures to low concentrations of pollutants and the possible risks over a longer period. Further, AI can help detect new chemicals of concern by mining existing datasets to reveal unseen relationships between chemical properties and toxicity. Additionally, AI can help identify the most vulnerable individuals, including those with specific genetic predispositions or exposure to certain environmental conditions (Cazzolla et al., 2020).

AI can be utilized to forecast the potential repercussions of pesticide exposure on local wildlife and human communities, for example. Researchers can pinpoint exposure biomarkers with the help of an AI algorithm that makes use of toxicogenomic data in order to forecast the long-term health impacts of pesticide residues present in water and soil sources. When the environmental data is analyzed in real-time, these AI systems provide early warnings of dangers such as a chemical spill by observing abnormal concentrations of chemicals (Banerjee et al., 2024).

A serious concern in the pharmaceutical industry is drug safety. The adverse drug reactions (ADRs) are among the leading causes of drug recalls, delays, and harm to patients. AI-backed toxicogenomics can help facilitate effective assessment and mitigation of drug toxicity risks. By using genomic data and AI algorithms, pharmaceutical companies can develop predictive models that inform about the potential value of adverse drug reaction and mitigate costly and harmful failures (Nagar et al., 2025).

Machine learning algorithms can help analyze genomic data from clinical trial samples with the goal to identify genetic variants predisposing patients to drug-induced toxicity. These models are also capable of predicting drug metabolism in different populations based on genetic variations, which can lead to the design of safe personalized treatment regimens. The introduction of innovative biomarkers for tracking the development of toxicity in patients during drug development and clinical testing is an exciting application of AI-led toxicogenomics (Vora et al., 2023).

AI is capable of identifying safer drugs for various diseases and diseases by analyzing chemical structures and their toxicity, and predicting. Artificial intelligence drug design has been successful in numerous cases ranging from clinical trials, FDA market approvals, ect. Such AI-based methods can not only increase the speed of drug development but also improve patient outcomes by identifying safer, more efficient therapeutic options.

## 2 Literature search methodology

A systematic literature search was conducted across multiple electronic databases, including PubMed/MEDLINE, Scopus, Web of Science, and Embase. The search was performed for articles published between January 2015 and June 2025. Search terms included combinations of: “toxicogenomics,” “artificial intelligence,” “machine learning,” “deep learning,” “toxicity prediction,” “predictive toxicology,” “environmental safety,” “pharmaceutical toxicity,” “adverse drug reactions,” “biomarker discovery,” and “multi-omics.” Boolean operators (AND, OR) were used to combine search terms.

Inclusion criteria were: (1) peer-reviewed original research articles or reviews; (2) studies applying AI/ML methods to toxicogenomics, toxicity prediction, or related omics data; (3) articles in English; and (4) studies reporting quantitative performance metrics. Exclusion criteria included: (1) conference abstracts and grey literature without peer review; (2) studies not reporting model performance; (3) articles unrelated to toxicology or AI/ML. An initial database search yielded approximately 1,240 records. After removal of duplicates and screening of titles/abstracts, 312 full-text articles were assessed for eligibility. A total of 62 studies were included in this review.

## 3 AI-driven toxicogenomics: applications, methods, and emerging technologies

Toxicogenomics plays an important role in the understanding of molecular toxicity. Traditional approaches for toxicity testing like animal testing can be expensive, time-consuming, and unethical. AI-based approaches have garnered promising solutions toward limitations. The implementation of AI and ML to toxicogenomics allows the prediction of toxicity, improvement of risk assessment and acceleration of discovery of biomarkers related to toxins (Liu et al., 2019).

AI-driven toxicogenomics leverages data from “omics” technologies, such as genomics, proteomics or metabolomics. These datasets frequently showcase complex patterns that human experts find challenging to decipher. Toxicity prediction on human and environmental health involves mining large datasets for pattern recognition and the use of algorithms such as support vector machines (SVM), random forests, and neural networks for data processing (Zhang et al., 2025b).

One of the most prominent applications of AI in toxicogenomics involves the development of predictive models to predict the toxicity of chemicals before clinical trials. AI algorithms analyze chemical structures and correlate them with genomic and transcriptomic data to predict adverse effects and assess safety (Sing et al., 2024).

Deep learning, one of the aritifical intelligence’s designated poses that was made possible recently with the ability to construct more complex models handling high-dimensional data has made the predictions more accurate (Sarker, 2021). Deep learning, a subfield of artificial intelligence, enables the construction of multi-layered neural architectures capable of handling high-dimensional genomic and chemical data, thereby substantially improving the accuracy and scalability of toxicity predictions compared to shallower models (Sarker, 2021).

These simulators can predict the biological effects of different chemicals, including potential adverse effects. This helps to mitigate animal testing and accelerate safety evaluation.

Detecting toxic pollutants with AI environmental safety (Raies and Bajic, 2016).

Through Environmental Toxicology, the application of AI-based toxicogenomics can be used to predict the impacts of pollutants such as pesticides, heavy metals as well as industrial chemicals. Artificial intelligence (AI) tools can leverage large datasets of environmental chemical exposures and genetic information to reveal how toxic exposures affect health.

They can offer insight into safety limits for chemical exposures while also guiding regulatory frameworks (Limbu et al., 2024).

In the pharmaceutical industry, the AI-based toxicogenomics plays a crucial factor in predicting the adverse drug reactions (ADRs). Drugs are withdrawn from the market primarily due to toxicity and it remains one of the biggest hurdles in drug research. Through genomic data, the AI models can identify the genetic variants that increase the risk of ADR which helps design safer and effective drugs (Dafniet and Taboureau, 2024; Tonoyan and Siraki, 2024).

In clinical trials, algorithms can analyze genomic data to find a match between drug and patient. In this way, at a genetic level, drugs can be predicted which suitability rate will be more-supported by the patient’s response. Such information is useful in generating personalized treatment regimens and minimizing ADRs among various population groups.

Moreover, AI can help to locate new biomarkers that can be used to identify toxicity during clinical development and monitoring the safety of drugs (Alkhoury et al., 2025).

## 4 Emerging AI technologies in toxicogenomics

Beyond conventional ML algorithms, a new generation of AI architectures is now being applied to toxicogenomics. Graph Neural Networks (GNNs) have demonstrated particular promise for molecular toxicity prediction, as they can directly encode molecular graphs and capture atom-level and bond-level features without requiring manual feature engineering. GNNs such as AttentiveFP and Directed Message Passing Neural Networks (D-MPNN) have achieved state-of-the-art performance on toxicity benchmarks including Tox21, ClinTox, and SIDER datasets.

Transformer-based architectures, originally developed for natural language processing, have been successfully adapted for molecular representation learning. Models such as ChemBERTa and MolBERT leverage self-supervised pre-training on large chemical databases and can be fine-tuned for toxicity prediction tasks. These approaches have shown improved generalization compared to traditional fingerprint-based methods. Foundation models and Large Language Models (LLMs) trained on vast biomedical and chemical text corpora are emerging as powerful tools for extracting structured toxicological knowledge, identifying novel toxicity pathways from literature, and supporting regulatory text analysis. Multimodal learning frameworks that integrate chemical structure data, gene expression profiles, imaging data, and clinical phenotypes simultaneously represent the next Frontier in AI-driven toxicogenomics, enabling holistic and mechanistically grounded toxicity prediction.

## 5 Discussion

Studies review that AI and ML are going to play an increasingly important role in toxicogenic progress, environmental toxicity prediction, and drug toxicity prediction. AI-based approaches have arrived on the scene as a more productive, ethical, and useful alternative to testing in toxicology. The studies show the applications, challenges and potential of AI and ML in toxicological sciences from a unique perspective.

In toxicogenomics research, Rahman et al., (2022) introduced ML-based methods to identify biomarkers from toxicogenomic data with promising results. This is a key step in connecting molecular interaction data to regulatory-relevant phenotypic endpoints. According to their findings, AI can help us predict how chemicals interact at the genetic level. This prediction will help with public health guidelines and regulatory laws. The study shows how to integrate AI with toxicogenomic data for the development of genomic biomarkers which could help in early toxicity prediction leading to reduction in animal testing and improvement in safety assessment efficiencies.

According to Jeong and Choi (2022), Artificial Intelligence and Machine Learning can contribute to predicting the environmental toxicity of toxic chemicals. There is a need to develop robust and effective AI-based prediction models for chemical management. According to the authors, one application of this type of AI will be to help reduce the risks due to exposure of hazardous chemicals. It should help identify toxicants and their toxicity to ecosystems and humans, among others. The research shows that the growing importance of AI for environmental solutions especially regulatory management and public health safety.

Tran et al. (2023) and Zhang et al. (2025a) mention AI and ML application for predicting toxicity of drug molecules. The studies highlight the continuous adaptation of predictive models in pharmaceutical research to find potential adverse drug reactions even before the initiation of clinical trials. According to Tran et al., recent progress and challenges in predicting drug toxicity have generated considerable interest. That’s because the genetic basis for adverse drug reactions (ADRs) is complicated. According to Zhang et al. the development of AI-driven models to predict toxicity in drugs using chemical structures and other data should be further built on. The use of AI in drug safety could lower late-stage failure costs and risks for drugmakers and patients.

Kim and Choi (2025) utilize ToxCast data to train AI models for toxicity prediction while highlighting the importance of explainable models in toxicology. This study shows how high-throughput screening data like ToxCast can help AI to predict chemical toxicity more accurately. According to the authors, regardless of the power of these models, transparency and interpretability remain a significant challenge for regulatory acceptance. It is essential for AI model predictions to be explicable to get developed into regulations because stakeholders would not trust the AI model to make decisions on their own without explanations.

In the paper of Lin and Chou (2022), an overall summary of AI and ML applications in toxicology is presented, describing how they are integrated into them. As per them, through AI, the improvement in efficiency of analysis is possible, while there is a greater understanding of how toxicity works. This review integrates various AI techniques which have made remarkable contributions to the field of toxicological sciences. It demonstrates the vast potential of AI to predict adverse effects from not only pharmaceutical but environmental chemicals too.

Read together, however, these studies diverge considerably in the datasets they rely upon, the way they validate their models, and the conclusions they reach, and these differences warrant closer scrutiny than a study-by-study reading conveys. Rahman et al. (2022) and Kim and Choi (2025) both draw on curated high-throughput resources—toxicogenomic expression profiles and ToxCast bioactivity data, respectively—yet only the latter treats interpretability as a precondition for regulatory use, whereas the former prioritises biomarker recovery over external validation. Jeong and Choi (2022) and Tran et al. (2023), by contrast, rely largely on retrospective benchmark datasets and internal cross-validation, an approach that tends to overstate performance relative to prospective or external testing; neither reports independent validation on chemically distinct compound sets, so their reported accuracies are not directly comparable. A limitation shared across all of these works is dataset imbalance and heterogeneity: toxic compounds are typically under-represented, and models trained on one platform or endpoint frequently fail to generalise to another, which makes headline performance figures difficult to weigh against one another. The studies also conflict on which architecture is preferable—Zhang et al. (2025a) and Tran et al. (2023) favour deep learning for its capacity to capture complex structure–toxicity relationships, while Kim and Choi (2025) and Lin and Chou (2022) caution that such gains often come at the expense of interpretability and are not consistently reproduced across endpoints. Viewed in this light, the reported performance differences appear to reflect dataset composition and validation design at least as much as any genuine algorithmic superiority, reinforcing the need for standardised benchmarks, balanced datasets, and prospective external validation before comparative claims can be regarded as robust.

### 5.1 Critical comparative analysis of AI approaches

Across the reviewed studies, several important patterns emerge that deserve critical examination. Classical ML approaches such as Random Forests and Support Vector Machines demonstrate strong performance on well-curated, balanced datasets, typically achieving AUC values of 0.75–0.88 on benchmark toxicity datasets (Tox21, ToxCast). However, these models depend heavily on hand-crafted molecular descriptors and fingerprints, limiting their ability to capture complex structural features. Deep learning architectures, in contrast, can learn hierarchical representations directly from raw input data and generally achieve superior performance (AUC 0.85–0.93) when sufficient training data is available. The trade-off, however, lies in interpretability: classical ML models such as Random Forests provide feature importance scores that regulatory scientists can examine, whereas deep neural networks function largely as black boxes.

A critical unresolved challenge across studies is the problem of dataset heterogeneity and imbalance. Toxicity datasets frequently exhibit severe class imbalance—with toxic compounds representing only 5%–20% of training samples in some datasets—which can artificially inflate accuracy metrics while masking poor sensitivity for rare toxic events. Several studies fail to report sensitivity and specificity alongside AUC, making cross-study comparisons unreliable. Furthermore, conflicting findings have been reported regarding which AI architecture is most suitable for different toxicological endpoints: while GNNs outperform traditional methods for acute toxicity prediction, multi-task deep learning models tend to perform better for mechanistic endpoint classification. These inconsistencies highlight the need for standardized benchmarking protocols. Table 1 below provides a systematic quantitative comparison of key AI methods and their reported performance across major studies.

**TABLE 1:** Systematic comparison of key AI studies in toxicogenomics.

Year	Application area	AI methods used	Key findings	Study
2022	Toxicogenomics, biomarker identification	Machine learning (ML)	Developed ML-based methods to identify biomarkers from toxicogenomic data, bridging to regulatory-relevant phenotypic endpoints	Rahman SM, Lan J, Kaeli D, Dy J, Alshawabkeh A, Gu AZ
2022	Environmental toxicity prediction	Artificial intelligence (AI), ML	Explores AI-based toxicity prediction models for environmental chemicals, emphasizing future directions for chemical management	Jeong J, Choi J
2023	Drug toxicity prediction	AI, ML	Discusses recent advances in AI and ML for predicting drug toxicity, outlining challenges and future perspectives	Tran TTV, Surya Wibowo A, Tayara H, Chong KT
2025	Drug toxicity prediction	AI, ML	AI-driven models for drug toxicity prediction, with a focus on challenges, future directions, and predictive methodologies	Zhang R, Wen H, Lin Z, Li B, Zhou X
2025	Toxicology, ToxCast data	AI, ML	Uses ToxCast data for AI-based toxicity prediction, highlights the need for explainable models in toxicology	Kim D, Choi J
2022	Toxicological sciences	AI, ML	Review of ML and AI applications in toxicology, providing a comprehensive overview of their integration into toxicological research	Lin Z, Chou WC

### 5.2 Challenges and future directions

AI-driven toxicogenomics demonstrates significant potential; however, several critical challenges remain. One of the primary limitations is data heterogeneity, arising from the diverse origins of genomic and toxicological datasets. For example, transcriptomic data are often affected by batch effects due to differences in experimental platforms and laboratory conditions, while metabolomic datasets frequently suffer from normalization inconsistencies and variability in analytical techniques. Such heterogeneity can compromise model robustness and generalizability if not properly addressed (Liu et al., 2019).

To overcome these limitations, advanced computational strategies such as federated learning and transfer learning have been proposed. Federated learning enables model training across decentralized datasets without requiring data sharing, thereby preserving data privacy while improving model generalization. Similarly, transfer learning allows models trained on large, well-annotated datasets to be adapted to smaller or domain-specific datasets, enhancing predictive performance in data-scarce scenarios. Incorporating these approaches could significantly strengthen AI-based toxicogenomic frameworks and improve their applicability across diverse datasets.

Additionally, despite high predictive accuracy, model interpretability remains a major concern. Many AI models, particularly deep learning architectures, function as “black boxes,” limiting their acceptance in regulatory contexts. Ensuring transparency through explainable AI (XAI) techniques is essential, especially for applications involving regulatory decision-making and safety assessment.

Furthermore, the regulatory integration of AI-driven toxicogenomics is still in its early stages. Clear guidelines and validation frameworks are required for the acceptance of AI models in safety assessment processes. Regulatory bodies must establish standardized protocols to ensure reliability, reproducibility, and trust in AI-generated predictions, thereby facilitating broader adoption in both environmental and pharmaceutical sectors.

### 5.3 Regulatory perspectives and frameworks for AI-Based toxicology

The regulatory acceptance of AI-driven toxicological models represents a significant and evolving challenge. The Organisation for Economic Co-operation and Development (OECD) has issued guidelines for the validation of (Quantitative) Structure–Activity Relationship [(Q)SAR] models (OECD Principle 5), which provide a foundational framework applicable to modern AI/ML toxicity models. These guidelines emphasize the need for a defined endpoint, an unambiguous algorithm, a defined domain of applicability, appropriate measures of goodness-of-fit and robustness, and a mechanistic interpretation where possible.

The U.S. Food and Drug Administration (FDA) has released guidance documents addressing the use of AI/ML in drug development and safety assessment, including the “Action Plan for AI/ML-Based Software as a Medical Device” and recommendations emphasizing algorithm transparency and real-world performance monitoring. The FDA’s Predictive Toxicology Roadmap further supports the integration of computational methods, including ML, for safety testing. Similarly, the European Medicines Agency (EMA) has initiated reflection papers on the use of AI in regulatory submissions, highlighting the importance of model explainability, uncertainty quantification, and prospective validation. Explainable AI (XAI) techniques—including SHAP (SHapley Additive exPlanations), lime (Local Interpretable Model-Agnostic Explanations), and attention visualization methods—are increasingly recognized as essential tools for satisfying regulatory transparency requirements. Widespread regulatory adoption will require the development of internationally harmonized validation frameworks, standardized performance reporting criteria, and formalized procedures for updating AI models as new toxicological data become available.

Several key research gaps and future directions emerge from this review. First, the field urgently needs large-scale, standardized, publicly accessible toxicogenomic databases that integrate multi-omics data with chemical exposure metadata and phenotypic outcomes. Existing resources such as ToxCast, DrugMatrix, and Open TG-GATEs provide valuable training data but differ substantially in design, platform, and annotation quality, complicating cross-dataset generalization. Second, despite promising results, very few AI toxicogenomic models have been prospectively validated on independent external datasets or confirmed with experimental studies. Prospective validation studies will be critical for establishing clinical and regulatory credibility. Third, the development of hybrid models that combine mechanistic biological knowledge (e.g., pathway networks, adverse outcome pathways) with data-driven ML approaches represents a highly promising but underexplored direction. Fourth, the integration of single-cell omics data with AI models opens entirely new opportunities for understanding cell-type-specific toxicity mechanisms at resolution not previously achievable. Addressing these gaps will require coordinated international collaboration among computational scientists, toxicologists, clinicians, and regulatory agencies.

## 6 Conclusion

AI-based toxicogenomics is a transformative tool in both environmental and pharmaceutical safety assessments. The combination of AI and high-throughput genomic technologies allows for the accurate prediction of toxicity, identification of biomarkers, and a deeper understanding of the molecular mechanisms behind toxic responses. As AI technologies continue to evolve, they promise to reduce reliance on traditional methods, improve risk assessments, and accelerate drug development, ultimately leading to safer products for both the environment and public health.
